# Subacute Subclinical Brain Infarctions after Transcatheter Aortic Valve Implantation Negatively Impact Cognitive Function in Long-Term Follow-Up

**DOI:** 10.1371/journal.pone.0168852

**Published:** 2017-01-05

**Authors:** Alexander Ghanem, Jonas Dörner, Leonie Schulze-Hagen, Andreas Müller, Marius Wilsing, Jan-Malte Sinning, Julian Lütkens, Christian Frerker, Karl-Heinz Kuck, Ingo Gräff, Hans Schild, Nikos Werner, Eberhard Grube, Georg Nickenig

**Affiliations:** 1 Department of Cardiology, Asklepios Klinik St. Georg, Hamburg, Germany; 2 Department of Cardiology, University Hospital Bonn, Germany; 3 Department of Radiology, University Hospital Cologne, Germany; 4 Department of Radiology, University Hospital Bonn, Germany; 5 Emergency Department, University Hospital Bonn, Bonn, Germany; Centro de Neurociencias de Cuba, CUBA

## Abstract

**Aims:**

To date every post-procedural cerebrovascular embolic event (CVE) is dreaded for its potential to accelerate cognitive decline after transcatheter aortic valve implantation (TAVI). This study differentiates the impact of acute (procedural) and post-acute cerebrovascular embolic events (CVEs) on cognitive performance.

**Methods:**

Magnetic resonance imaging (MRI) before, early and late after TAVI was performed to quantify embolic burden. Quantification of diffusion- and T1-weighted lesions, as well as white-matter and total brain volumes, as well as cognitive function testing (MMSE) were assessed in 28 patients with a medium follow-up period of 34 months.

**Results:**

Procedural diffusion-weighted lesions were observed in 17 patients (61%), but demonstrated locoregional remnants only in a minority of patients in long-term follow-up (6.5%). Acute CVEs did not impact the trajectory of late silent brain infarctions (SBI), white-matter hyperintensities, and cerebral atrophy. Functionally, early CVEs did not affect cognitive function. In contrast, patients with “new” SBIs after TAVI had a trend to cognitive deterioration in long-term follow-up (“new”SBI: MMSE -1.4 / no “new”SBI: MMSE +1.5, p = 0.067). Interestingly, only a fraction of these “new” SBIs evolved from procedural CVEs (22.2%).

**Conclusions:**

Aquired SBIs after TAVI, but not DW-CVE *per se* are associated with functional impairment long-term after TAVI. In the context of subacute thrombosis seen in TAVI prostheses, these findings set the stage for tailored stroke prevention and comprehensive surrogate endpoint definitions in neuroprotective trials.

## Background

Transcatheter aortic valve implantation (TAVI) is a valid therapeutic option for the treatment of aortic valve stenosis in patients at high risk for surgery [1]. The benefits, however, are mitigated by the occurence of (post-)procedural cerebrovascular embolic events (CVEs). The great majority of patients (63–84%) demonstrate clinically silent CVEs in diffusion-weighted magnetic resonance imaging (DW-MRI), disabling and non-disabling strokes are rare (1–3%) events [2–4]. The cumulated risk of CVEs remains elevated in the first post-procedural year, most conceivably associated with thrombogenicity of the device and the new-onset of atrial fibrillation [5, 6]. Since the incidence of CVEs in DW-MRI is widely utilized as a surrogate parameter of stroke risk, e.g. in neuroprotective approaches, and TAVI is more frequently utilized in younger, intermediate-risk patients, the contributions of silent microembolism to the functional and morphological cerebral outcome are of paramount importance.

The occurence and accumulation of silent brain infarctions (SBIs) in T2-weighted MRI was shown to be correlated with cognitive decline in the large-scale Rotterdam-Scan-Study [7]. Knowing this, CVEs in DW-MRI after TAVI are dreaded for their potential adverse impact on cerebral morphology and consecutive impairment of cognitive performance. However, the functional impact of CVEs in DW-MRI after surgery with extracorporal circulation remains controversially discussed, with mixed results across studies [8, 9]. In the context of TAVI, the morphological proof of CVEs in DW-MRI has not been shown to be causally connected to functional outcomes. CVEs were neither associated with negative cognitive outcomes, nor with dependence in daily activities or mortality [10, 11]. These observations were recently flanked by a meta-analysis, which has shown that the risk of cognitive decline after cardiovascular procedures is low, despite the knowledge on the interventions’ high cerebral embolic burden [12]. In all, the functional impact and long-term prognosis of silent CVEs seen with DW-MRI is not elucidated yet.

Evidence of SBIs is not uncommon in the elderly and increasing with age [13]. Besides SBIs, adverse cerebral remodelling measured by repetitive MRI encompasses also the less specific entities of white-matter hyperintensities (WMH) and cerebral atrophy (CA) [14]. Large observational studies demonstrated the association of SBIs with both, subclinical heart failure, as well as impaired cognitive outcome in patients without overt cerebrovascular events [7, 15, 16]. It was further demonstrated, that WMH-burden, especially a significant increase over time, as well as significant atrophy of brain tissue are both associated with cognitive deterioration [17]. Early signs of adverse cerebral remodelling are correlated with increased long-term risk for stroke and death [18]. However, only a fraction of TAVI-related DW-CVEs resulted in SBIs in T2-weighted MRI after three months [2]. Hence, the dreaded causality of TAVI-related acute CVEs in DW-MRI and consecutive aggravation of adverse remodelling in long-term trajectories remains elusive.

We hypothesized, that in long-term observations specifically the acquisition of SBIs (and not any DW-CVEs *per se*) affects brain morphology and cognition. Hence, we investigated the differential impact of early and later CVEs on functional and morphological outcome in a prospective study with long-term follow-up of cognitive function assessment and cerebral imaging with MRI.

## Methods

All patients scheduled for TAVI between October 2009 and September 2010 were screened for inclusion, aiming at a minimum follow-up period of 30 months. Indication for TAVI was in concordance with the recent consensus statement [19]. Inclusion and exclusion criteria are depicted in the methods supplement (S1 Methods). The study protocol was approved by the local institutional review board and followed the Declaration of Helsinki guidelines. Written informed consent was obtained from all patients.

### Study design

Baseline examinations (BL) were performed within five days prior TAVI. After careful investigation of medical history, the mortality risk was estimated by both, the logistic EuroSCORE and the risk score of the Society of Thoracic Surgeons (Society of Thoracic Surgeons score: mortality). Twelve-lead surface ECG, serological and hematological analyses, transthoracic and transesophageal echocardiography, color-coded duplex sonography of the extracranial carotid arteries, and coronary angiography were conducted. Clinical examinations according to the National Institutes of Health Stroke Scale (NIHSS) were performed. Furthermore, the repeatable mini-mental state exam for the assessment of general cognitive function (MMSE) was conducted by a trained neuropsychological research fellow. The MMSE is a valid neuropsychological testing battery, which is suited for the assessment of cognition in elderly subjects. We additionally assessed frailty, quality of life, instrumental activities of daily living, and mood using the following validated scores and questionnaires: The Edmonton frail scale scores of ≤3 and >7 indicate a lower (odds ratio: 0.3) and excessive risk (odds ratio: 5.0) of having a complication after surgery, respectively. The health-related short form allows the assessment of mental and physical health–related status on a scale ranging from 0 to 100. The Lawton instrumental activities of daily living score displays function in daily activities, with higher values reflecting better function; a value >52 has previously been used to determine independent lifestyle. The geriatric depression score is a 30-item report, which displays patient mood on a scale ranging from 0 to 30, with a value <10 reflecting lack of depressive symptoms. The occurrence of systemic inflammatory response syndrome (SIRS) was defined as fulfilling ≥2 of the following 4 criteria: body temperature <36.0°C or >38.0°C, heart rate >90 bpm, respiratory rate >20 breaths/min, or PaCO_2_ <32 mm Hg, leukocyte count >12 or <4 (10^9^/L) 1, 6, 24, 48, or 72 hours after TAVI. Preprocedural cerebral DW-MRI was performed within three days prior TAVI. First postprocedural investigations (FU1) were performed within three days after TAVI, second follow-up investigations (FU2) were performed after at least 30 months.

### Cerebral magnetic resonance imaging

Cerebral MRI was performed using a 1.5-T whole body system (Philips Medical Systems, Best, The Netherlands). The scanning protocol comprised transversal T1-weighted, transversal T2-weighted, transversal Fluid Attenuated Inversion Recovery (FLAIR) as well as transversal and coronal diffusion-weighted imaging, respectively. A single trained board-certified neuroradiologist who was unaware of the patients’ history of stroke and transient ischemic attack scored and quantified lesions on the baseline and the two follow-up MRIs with respect to their location and size. As described previously, we regarded the progressions of WMH >1.5%/year and CA >1%/year as significant. Detailed imaging protocols and method of data analysis are presented in the methods supplement (S1 Methods).

### Transcatheter aortic valve implantation

TAVI was performed in spontaneously breathing patients in deep sedation using midazolam, propofol, and fentanyl. Details are described in the methods supplement (S1 Methods). In a subset of patients, valvuloplasty, rapid ventricular pacing, and postdilatation were omitted at the discretion of the operator. This approach has been described recently and was abbreviated as direct TAVI.

### Statistical analyses

Continuous variables are presented as mean±SD if normally distributed and as median (interquartile range) if not normally distributed. Categorical variables are given as frequencies and percentages and were compared by χ^2^ statistics or Fisher exact test. Continuous variables were tested for differences by means of a 2-sided, unpaired Student t test for comparison between groups and with a 2-sided, paired Student t test for intragroup comparison. Nonparametric testing (Mann–Whitney test) was performed where indicated. A value of P<0.05 was considered statistically significant. Analyses were conducted with SPSS Statistics version 17.0.0 (22.0.0) (SPSS Inc, Chicago, IL).

## Results

**Fig 1:**
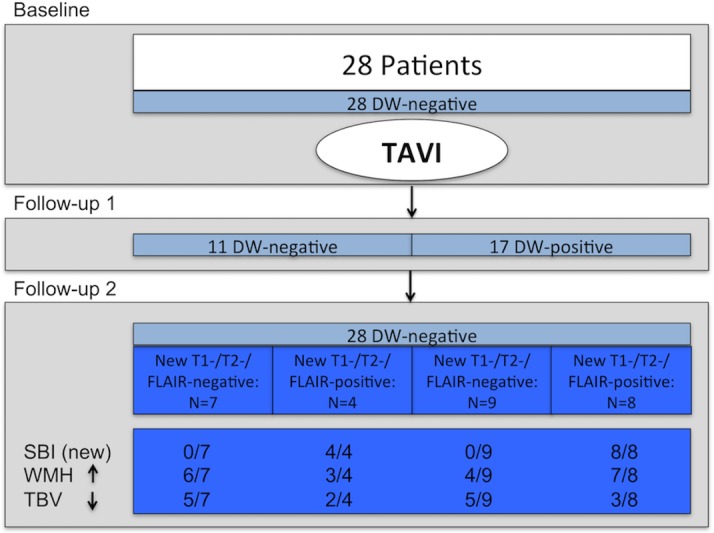
Morphological cerebral outcome after TAVI. The majority of patients (60.7%) had acute lesions in cerebral diffusion-weighted magnetic resonance imaging (DW-MRI) after TAVI. However, there was no association of these acute events (DW-CVEs) and the incidence of SBIs after long-term follow-up. Moreover, DW-CVEs neither aggravated the progression of white matter hyperintensities (> 1.5% / year), nor the progression of cerebral atrophy (> 1% / year). TAVI–Transcatheter aortic valve implantation, DW–diffusion-weighted, CVE–cerebrovascular event, MRI–magnetic resonance imaging, WMH–white matter hyperintensities, TBV–total brain volume, FLAIR—Fluid Attenuated Inversion Recovery.

Patient baseline characteristics were comparable to previously published data (see Table 1). Twenty-eight patients were followed-up for a mean of 2.8 years (33.6 months). During the follow-up period, no clinically aparent cerebrovascular accident was observed. At baseline (BL) no patient had CVEs in DW-MRI. Ten patients (35.7%) had 14 SBIs (range 1–3 / patient). At FU1, 17 of 28 patients (60.7%) demonstrated 61 acute post-procedural CVEs in DW-MRI (range 1–14 / patient). At FU2, all patients were negative for DW-CVEs. Notably, 17 of 28 patients demonstrated 32 SBIs (60.7%, range 1–5 / patient), 12 of these patients had 18 “new” SBIs at FU2 (Fig 1).

**Table 1:** Clinical baseline characteristics.

**Clinical Data @ BL**	
N	28
Age, years ±SD	80 ±6
Male, n (%)	14 (50)
Body mass index, kg/m^2^ ±SD	25.6 ±5.0
Log EuroSCORE, % ±SD	24.1 ±17.9
STS score: mortality, % ±SD	7.5 ±6.9
STS score: permanent stroke, % ±SD	3.0 ±1.8
Peak-to-peak gradient, mmHg ±SD	48 ±22
Ejection fraction, % ±SD	49 ±15
NYHA class ±SD	3 ±1
Minimental state examination score @ BL	26.6 ±2.8
Minimental state examination score @ FU2	26.7 ±3.6
**Comorbidities**	
Hypertension, n (%)	26 (93)
Diabetes, n (%)	4 (14)
Smoking, n (%)	6 (21)
Dyslipidemia, n (%)	17 (61)
Creatinine, mg/dl ±SD	1.2 ± 0.4
Glomerular fibrilation rate, ml/min ±SD	55 ± 9
Hemodialysis, n (%)	0 (0)
Atrial fibrillation or flutter, n (%)	8 (29)
CHADS_2_-Score ±SD	2.5 ± 1.2
Prior stroke or TIA, n (%)	7 (25)
PVD, n (%)	10 (36)
Coronary artery disease, n (%)	14 (50)
Prior myocarial infarction, n (%)	4 (14)
Prior PCI, n (%)	8 (29)
Prior CABG, n (%)	1 (4)
**Procedural characteristics**	
Procedure time, min ±SD	98 ± 54
Direct TAVI without predilatation, n (%)	8 (29)
Corevalve 23/26/29/31 mm, n	0/9/12/2
Edwards-Sapien 23/26 mm, n	2 /1
Symetis 23 mm, n	2
Post-dilatation, n (%)	7(25)
Rapid pacing runs, n (%)	1.1 ±1.0
**Post-procedural characteristics @ FU 1**	
SIRS, n (%)	8 (29)
Minor bleeding, n (%)	10 (36)
Major bleeding, n (%)	1 (4)
Stroke, n (%)	0 (0)
Evidence of embolic events in DW-MRI, n(%)	17 (61)
**Medication @ FU 2**	
Acetylsalicylic acid, n (%)	21 (75)
Clopidogrel hydrogen sulphate, n (%)	3 (11)
Beta-blocker, n (%)	24 (86)
Statin, n (%)	17 (61)
AT1 antagonist, n (%)	9 (32)
ACE inibitor, n (%)	14 (50)
Diuretics, n (%)	22 (79)

**Fig 2:**
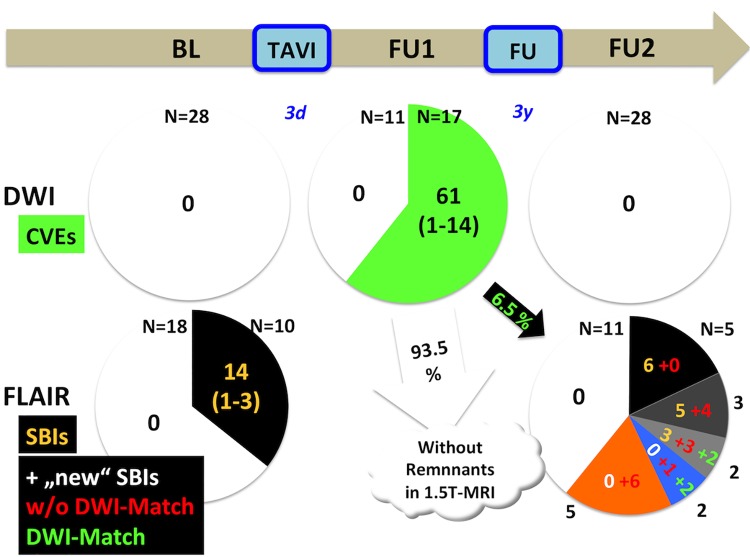
Trajectories of acute and subacute cerebral lesions after TAVI. Patients underwent MRI prior (left), early (mid) and late (right) after TAVI. Above: The majority of patients demonstrate cerebral embolic events early after TAVI. No spontaneous lesions were observed prior and late after TAVI. Below: Ten out of 28 patients undergoing TAVI had 14 (old, non-procedural) silent brain infarctions (SBIs) in MRI (left). After long-term follow-up, seven patients without "old" SBIs revealed "new", post-procedural events. Notably, only four out of 18 "new" SBIs had an early procedural correlate in diffusion-weighted imaging (right).

**Fig 3:**
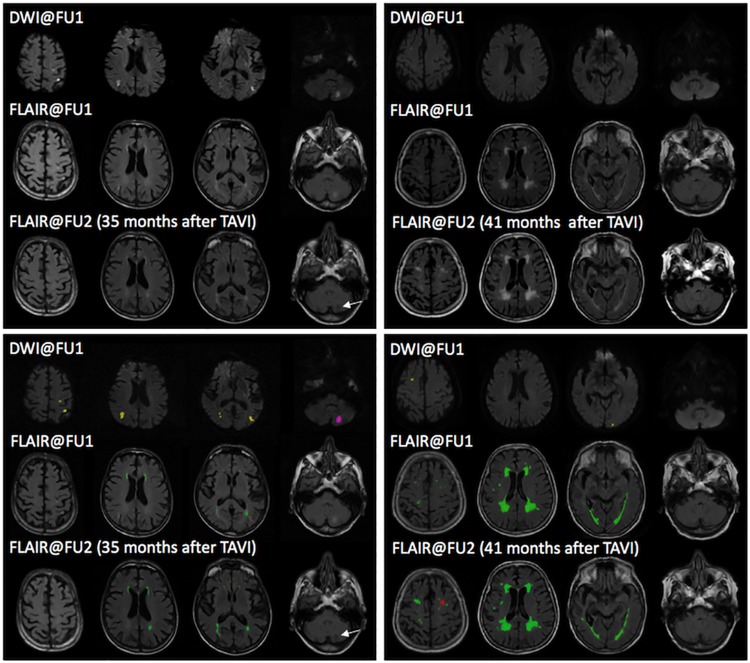
Representative MRIs of patients after TAVI. Above: Representative MRI data sets of two patients showing raw data (upper row) and overlay images (lower row). Images were acquired early (DWI@FU1 and FLAIR@FU1) and late (FLAIR@FU2) after TAVI. Below: On overlay images, yellow lesions display acute procedural CVEs which vanished without detectable remnants @FU2, purple lesions display acute procedural DW-CVEs with scarring @FU2, red lesions display “new”, non-procedural SBIs @FU2 in a location where previously no DW-CVEs were present, and green lesions display WMHs. DWI@FU1 after TAVI shows procedural CVEs in both patients. FLAIR imaging acquired at FU1 shows mild (left) and severe (right) WMH-load. However, both demonstrated only slight increase throughout the follow-up period of 35 and 41 months, respectively. The left patient developed a parenchymal defect in the left cerebellum (FLAIR@FU2) in the same location of the DW-CVE at FU1, indicating scarring of a procedural CVE. In contrast, the right patient developed an infarct-like lesion in the left frontal lobe in a location where no DW-CVE was previously recognized at FU1 (“new”, non-procedural SBI). Only a fraction of peri-procedural, acute DW-CVEs resulted in detectable infarct-like lesions in follow-up scans (arrow). DWI–Diffusion-weighted imaging, CVE–Cerebrovascular event, WMH–White matter hyperintensities, SBI–Silent brain infarction, FLAIR—Fluid Attenuated Inversion Recovery, FU–Follow-up, TAVI–Transcatheter aortic valve implantation.

Baseline characteristics were distributed equally with respect to procedural events (see Table 2). Also, none of the baseline characteristics was associated with the occurrence of “new” SBIs at FU2. In particular, patients with “new” SBIs have equally distributed procedural events in DW-MRI (see Table 3). Seven patients (25%) had their first-ever SBI during the follow-up period. Of those, two had a loco-regionally “matched”, procedural event. On the contrary, two patients had no corresponding post-procedural CVE in DW-MRI. So, the majority of “new” SBIs were not causally related to the procedure itself. Of 61 DW-lesions obtained in FU1, only a fraction (n = 4, 6.5%) had a visible residuum in T1- and T2-weighted MRI at FU2, whereas the majority (93.5%) was not demarkated. Of the 18 new SBIs at FU2, only 4 (22.2%) were offsprings and causally related to an early, procedural DW-positive event at FU1 derived from the procedure itself. For details see Fig 2, two representative MRI-datasets are displayed in Fig 3.

**Table 2:** Univariate analyses of clinical baseline characteristics dichotomized for the occurence of a new procedural DWI event at follow-up 1.

Clinical Data	DWI Lesion Present @FU1 (n = 17)	DWI Lesion Absent @FU1 (n = 11)	p-Value
Age, years ±SD	81 ±6	79 ±6	0.55
Male, n (%)	10 (59)	4 (36)	0.44
Body mass index, kg/m^2^ ±SD	24.8 ±5.2	26.9 ± 4.5	0.27
Log EuroSCORE, % ±SD	22.9 ±17.1	25.8 ± 19.8	0.69
STS score: mortality, % ±SD	7.8 ±7.7	7,0 ± 5,7	0.75
STS score: permanent stroke, % ±SD	2.7 ±0.8	3,5 ± 2,7	0.24
Peak-to-peak gradient, mmHg ±SD	46 ±21	52 ± 25	0.45
Ejection fraction, % ±SD	51 ±15	46 ± 15	0.40
NYHA class ±SD	3 ±1	3 ± 0	0.39
Minimental state examination score BL	26.1 ±3.2	27.3 ±2.3	0.33
Minimental state examination score FU2	26.2 ±4.4	27.5 ±2.2	0.40
**Comorbidities**			
Hypertension, n (%)	17 (100)	9 (82)	0.15
Diabetes, n (%)	2 (12)	2 (18)	1.00
Smoking, n (%)	4 (24)	2 (18)	1.00
Dyslipidemia, n (%)	10 (59)	7 (64)	1.00
Creatinine, mg/dl ±SD	1.1 ± 0.2	1.3 ± 0.6	0.35
Glomerular fibrilation rate, ml/min ±SD	53.8 ± 8.9	55.9 ± 9.8	0.19
Hemodialysis, n (%)	0 (0)	0 (0)	-
Atrial fibrillation or flutter, n (%)	5 (29)	3 (27)	1.00
CHADS_2_-Score ±SD	2 ±1	3 ±1	0.18
*Prior stroke or TIA*, *n (%)*	*1 (6)*	*6 (55)*	*0*.*01*
PVD, n (%)	4 (24)	6 (55)	0.13
Coronary artery disease, n (%)	10 (59)	4 (36)	0.44
Prior myocarial infarction, n (%)	2 (12)	2 (18)	1.00
Prior PCI, n (%)	6 (35)	2 (18)	0.42
Prior CABG, n (%)	1 (6)	0 (0)	1.00
**Procedural characteristics**			
Procedure time, min ±SD	109 ± 66	80 ± 20	0.17
Direct TAVI without predilatation, n (%)	12 (71)	8 (73)	1.00
Corevalve 23/26/29/31 mm, n	0/5/7/1	0/4/5/1	-/1.0/1.0/1.0
Edwards-Sapien 23/26 mm, n	1/1	1/0	1.0/1.0
Symetis 23 mm, n	2	0	0.50
Post-dilatation, n (%)	3 (18)	4 (36)	0.38
Rapid pacing runs, n (%)	1.2 ±1.2	0.8 ±0.6	0.86
**Post-procedural characteristics**			
SIRS, n (%)	5 (29)	3 (27)	1.00
Minor bleeding, n (%)	6 (35)	4(36)	1.00
Major bleeding, n (%)	1 (6)	0 (0)	1.00
Stroke, n (%)	0 (0)	0 (0)	-
**Medication**			
Acetylsalicylic acid, n (%)	12 (71)	9 (82)	0.67
Clopidogrel hydrogen sulphate, n (%)	3 (18)	0 (0)	0.26
Beta-blocker, n (%)	15 (88)	9 (82)	1.00
Statin, n (%)	10 (59)	7 (64)	1.00
AT1 antagonist, n (%)	5 (29)	4 (36)	1.00
ACE inibitor, n (%)	8 (47)	6 (55)	1.00
Diuretics, n (%)	14 (82)	8 (73)	0.65

**Table 3:** Univariate analyses of clinical baseline characteristics dichotomized for the occurence of new SBIs at follow-up 2.

Clinical Data	“New”SBI present @FU2 (n = 12)	“New”SBI absent @FU2 (n = 16)	p-Value
Age, years ±SD	81 ±6	80 ±6	0.54
Male, n (%)	7 (58)	7 (44)	0.70
Body mass index, kg/m^2^ ±SD	25.3 ±4.5	25.9 ±5.5	0.76
Log EuroSCORE, % ±SD	23.6 ±18.6	24.4 ±18.0	0.91
STS score: mortality, % ±SD	7.1 ±5.4	7.8 ±8.0	0.81
STS score: permanent stroke, % ±SD	2.5 ±1.0	3.4 ±2.2	0.23
Peak-to-peak gradient, mmHg ±SD	40.3 ±22.8	54.4 ±20.7	0.10
Ejection fraction, % ±SD	50.0 ±15.2	48.4 ±15.5	0.78
NYHA class ±SD	2.9 ±0.3	2.9 ±0.7	0.92
Minimental state examination score BL	27.1 ±2.7	26.2 ±3.0	0.43
Minimental state examination score FU 2	25.7 ±0.3	27.7 ±1.9	0.17
**Comorbidities**			
Hypertension, n (%)	11 (92)	15 (94)	1.00
Diabetes, n (%)	1 (8)	3 (19)	0.61
Smoking, n (%)	2 (17)	4 (25)	0.67
Dyslipidemia, n (%)	8 (67)	9 (56)	0.71
Creatinine, mg/dl ±SD	1.1 ±0.2	1.2 ±0.6	0.49
Glomerular fibrilation rate, ml/min ±SD	59.7 ±8.7	56.5 ±15.1	0.52
Hemodialysis, n (%)	0 (0)	0 (0)	-
Atrial fibrillation or flutter, n (%)	3 (25)	5 (31)	1.00
CHADS_2_-Score ±SD	2.3 ±0.8	2.8 ±1.4	0.27
Prior stroke or TIA, n (%)	1 (8)	6 (38)	0.18
PVD, n (%)	3 (25)	7 (44)	0.43
Coronary artery disease, n (%)	5 (42)	9 (56)	0.70
Prior myocarial infarction, n (%)	3 (25)	1 (6)	0.29
Prior PCI, n (%)	2 (17)	6 (38)	0.40
Prior CABG, n (%)	1 (8)	0 (0)	0.43
**Procedural characteristics**			
Procedure time, min ±SD	79.1 ±33.2	111.6 ±63.1	0.12
Direct TAVI without predilatation, n (%)	3 (25)	5 (31)	1.00
Corevalve 23/26/29/31 mm, n	0/4/5/2	0/5/7/0	-/1.00/1.00/0.18
Edwards-Sapien 23/26 mm, n	0/0	2/1	0.49/1.0
Symetis 23 mm, n	1	1	1.00
Post-dilatation, n (%)	1 (8)	6 (38)	0.18
Rapid pacing runs, n (%)	0.9 ±0.7	1.2 ±1.2	0.50
**Post-procedural characteristics**			
SIRS, n (%)	3 (25)	5 (31)	1.00
Minor bleeding, n (%)	2 (17)	8 (50)	0.11
Major bleeding, n (%)	0 (0)	1 (6)	1.00
Stroke, n (%)	0 (0)	0 (0)	-
Evidence of embolic events in DW-MRI, n(%)	8 (67)	9 (56)	0.71
**Medication @FU2**			
Acetylsalicylic acid, n (%)	8 (67)	13 (81)	0.42
Clopidogrel hydrogen sulphate, n (%)	1 (8)	2 (13)	1.00
Beta-blocker, n (%)	10 (83)	14 (88)	1.00
Statin, n (%)	6 (50)	11 (69)	0.44
AT1 antagonist, n (%)	2 (17)	7 (44)	0.22
ACE inibitor, n (%)	7 (58)	7 (44)	0.70
Diuretics, n (%)	10 (83)	12 (75)	0.67

White-matter hyperintensities (WMH) were present in all patients (n = 28, 100%) at baseline. The mean WMH-burden at BL was 12.8 +/-9.1 ml (Median: 13.5, interquartile range: 4.2–19.6), accounting for 0.1–2.8% of the total intracranial volume (mean 0.9 ± 0.7%). The mean WMH-burden at FU2 increased to 16.4 +/-11.9 ml (Median: 15.3, interquartile range: 6.3–23.1, p < 0.001), accounting for 0.1–3.2% of the total intracranial volume (mean 1.2 ± 0.9%) and resulting in a mean increase of WMH-burden of 1.3 ml/year. Twenty-four of the 28 patients demonstrated an increase of WMH-volume exceeding 1.5% /a, indicating accelerated WMH-formation. Patients with and without accelerated WMH-formation had no significant differences with respect to the occurrence of procedural CVEs at FU1, and baseline characteristics. The occurence of new SBIs at FU2 was associated with a slight, but not significant increase of total WMH-volume (“new” SBIs: WMH-volume +18.3%/a; no “new” SBIs: WMH-volume + 12.1%/a; p = 0.28).

The mean total brain volume (TBV) at BL was 1183 +/-119 ml (Median: 1185, interquartile range: 1108–1255), the mean TBV at FU2 decreased to 1114 +/-112 ml (Median: 1092 ml, interquartile range: 1040–1199 ml). Eighteen of the 28 patients demonstrated a decrease of TBV exceeding 1%/a, indicating accelerated brain atrophy. Patients with and without accelerated brain atrophy had no significant differences with respect to the occurrence of DW-CVEs at FU1, and baseline characteristics. The occurence of new SBIs at FU2 was not associated with a significant decrease of TBV (“new” SBIs: TBV –2.0%/a; no “new” SBIs: TBV– 2.5%/a; p = 0.59).

**Fig 4:**
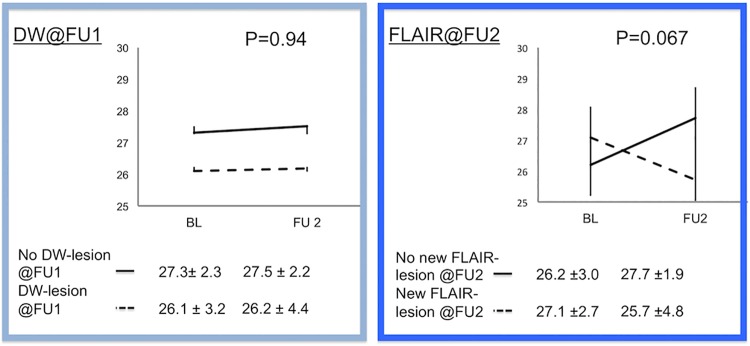
Functional outcome after TAVI. Cognitive outcome was not influenced by the occurence of new events in diffusion-weighted magnetic resonance imaging (DW-MRI) early after TAVI (FU1, left side). However, patients with “new”, non-procedural SBIs (confirmed in FLAIR-imaging) during long-term follow-up after TAVI have a trend towards cognitive decline as compared to patients without. DW–Diffusion-weighted, FU–Follow-up, FLAIR—Fluid Attenuated Inversion Recovery MMSE–Mini-mental state exam.

The mean MMSE scores of the entire cohort at baseline and FU2 demonstrated no significant difference and were 26.6 +/-2.9 and 26.7 +/-3.7, respectively (p = 0.88). Neither the occurrence of WMH-increase over time, nor the TBV-decrease at FU2, and distinctly not DW-CVEs at BL impacted MMSE-trajectory negatively. In contrast, the presence of “new” SBIs at FU2 negatively impacted MMSE-trajectories during follow-up (“new”SBI: MMSE -1.4 / no “new”SBI: MMSE +1.5, p = 0.067), indicating a negative functional effect of new SBIs, but not other imaging parameters on cognitive function (Fig 4).

## Discussion

This is the first report on morphological and functional long-term outcome of CVEs after TAVI. In spite of the raised concerns on the high incidence of procedural, silent cerebral embolism, this study adds several new insights: Firstly, only a fraction of the cumulative cerebral embolic burden throughout the first three years after TAVI is related to the procedure itself. More than 95% of the new SBIs in follow-up were not related to the TAVI-procedure itself. Secondly, DW-CVEs had no impact on late adverse cerebral morphological outcome. The incidences of SBIs and brain atrophy, as well as the trajectories of WMH were independent of the occurence of procedural DW-CVEs. Ultimately, we observed the trend, that new SBIs, but not DW-CVEs, negatively impacted cognitive outcome. Hence, the dreaded long-term effects of DW-CVEs on brain morphology and function could not be objectified after a median of three years of follow-up.

Longitudinal imaging studies on the fate of procedural embolism are of particular interest, since the dreaded risk for future stroke and cognitive deterioration is limited to the presence of SBIs. In the large-scale Rotterdam Scan Study (> 1000 normal elderly, mean age 71, followed over 3.6 years), the presence of SBIs on the baseline MRI was associated with worse performance on neuropsychological tests and a steeper decline in global cognitive function. Interestingly, when participants with SBIs at baseline were subdivided into those with and those without additional infarcts at follow-up, the decline in cognitive function was restricted to those with additional silent infarcts [7]. Recent meta-analyses demonstrated, that SBIs of small volume < 3mm were independent predictors of later stroke and mortality [18]. The protocol allowed us to differentiate the effects of the short, but intense embolic “rainstorm”during the procedure from the long-lasting, continuous embolic “drizzle”based on the aquired risk factors (NOAF, valve thrombosis). Longitudinal study protocols utilize cerebral MRI as a “biological event recorder”integrating and cumulating all procedural and post-procedural cerebral events throughout the follow-up period. Despite the dreaded impact of procedural embolism, only a small fraction (6.5%) of TAVI-induced DW-CVEs resulted in SBIs in long-term follow-up. This finding objectifies the very limited part of the peri-procedural embolic burden on the total structural cerebral remodelling during follow-up. We observed that > 85% of the acquired SBIs after TAVI were not related to the embolism during the procedure itself. Hence, in future studies longitudinal imaging protocols could take post-embolic cerebral remodeling into account. As in experimental research, early DW- and follow-up T2-weighted MRI might indicate the area at risk and the infarct scar, respectively.

With advancing age, WMH-burden increases [14], and total brain volume decreases (cerebral atrophy). The extent of volume changes has been associated with declining scores in the modified MMSE and the Digit Symbol Substitution Test, as well as with incident MCI, dementia, and death [20, 21]. More recent studies demonstrated that progression of WMH is a better predictor of persistent cognitive impairment than baseline WMH-burden alone [17]. The group of Silbert et al. have further shown that in a cohort of octogenarians greater WMH progression rate is associated with increased rate of decline on gait and memory function in cognitively intact elderly [17]. WMH acceleration may directly result in cognitive decline years later. Interestingly, the acceleration in WMH volume increase occurred 3.7 years before the onset of cognitive deterioration [17]. Hence, WMH-Progression was able to predict cognitive impairment in a very early phase. Based on the cut-off values in neurodegenerative disease, a loss of more than 1% / year total brain volume was set as significant for brain atrophy. In a large-scale observational study, total brain volume loss was significantly associated with cognitive impairment. Erten-Lyons and collegues suggest that there may be other factors contributing to brain atrophy, e.g. cardiovascular risk factors [22]. We therefore utilized these two dynamic, intraindividual parameters of cerebral remodelling to sensitively investigate the dreaded adverse impact of DW-CVEs. Despite the advanced age and comorbid conditions of our study population, DW-CVEs had neither impact on the course of WMH-burden and cerebral atrophy after TAVI in our cohort.

In a functional follow-up study, the occurence of DW-CVEs after TAVI did not impact cognitive trajectories for up to two years [10]. Further, a recent meta-analysis objectified the low risk of cognitive impairment after cardiovascular interventions [12]. This is of particular interest in spite of the known high emboligeneic burden of these procedures with a DW-CVE-risk of up to 84%. However, DW-CVEs were not associated with worse outcomes with respect to self-sufficiency in daily activities, quality of life, cognitive performance and trajectories, as well as mortality after TAVI [9, 11]. Here, new SBIs but not DW-CVEs negatively impacted funtional outcome. Although statistically the results missed the level of significance, the patients with SBIs at FU2 decreased in MMSE score by 1.4 points, whereas patients without new SBIs improved by 1.5 points. These results are not only reasonable since only 1 in 14 DW-lesions resulted in local adverse remodelling and SBI formation. Also, the remainder (77.8%) of embolic events cumulated throughout the follow-up period and were not local remnants of the acute DW-CVEs. Beside NOAF, valvular thrombosis was recently published as a potential mechanism of subacute stroke after TAVI [6]. Pache and collegues described early hypo-attenuated leaflet thickening, which was reversed by oral anticoagulation. This transient thrombosis of valvular leaflets is a mechanism explaining subacute events and could be the reason for the observed rate of „new”SBIs. Hence, thrombus formation might further explain the high proportion of patients experiencing their „first”SBI after TAVI. As additional neuroprotective approach, oral anticoagulants are currently investigated for their value in prevention of subacute stroke after TAVI.

### Limitations

This is a monocentric observation of a small patient cohort, hoever the first to provide long-term imaging follow-up. Mini-mental state exam is a coarse test which has its known limitations. But the MMSE is a valid tool, which can be set into context with a plethora of published datasets. Cerebral imaging was mostly performed with 1.5, and not 3 Tesla. Not all lesions attributed to CVD-BI on MRI are in fact caused by vascular injury. However, due to the lack of long-term imaging investigations, currently we are not able to specifically differentiate embolic from degenerative disease. Although it is known, that the embolic risk is highest during the first month after TAVI, the exact timepoint of post-procedural SBIs between FU1 and FU2 remains unclear and needs to be further elucidated in future trials.

### Conclusions

In all, this pilot study tones down the dreaded impact of silent, procedural events seen in DW-MRI on functional and morphological long-term outcome in a high-risk population. Functional long-term prognosis seems to be determined by the accumulation of SBIs and should be considered in future studies of neuroprotective approaches.

## Supporting Information

S1 Methods Supplementary Methods. Transfemoral aortic valve implantation Cranial MRI and imaging analysis.
(DOCX)
