# Correction: Subacute Subclinical Brain Infarctions after Transcatheter Aortic Valve Implantation Negatively Impact Cognitive Function in Long-Term Follow-Up

**DOI:** 10.1371/journal.pone.0172186

**Published:** 2017-02-09

**Authors:** The PLOS ONE Staff

The second and seventh authors’ names are spelled incorrectly. The correct names are: Jonas Doerner and Julian Luetkens.

The correct citation is: Ghanem A, Doerner J, Schulze-Hagen L, Müller A, Wilsing M, Sinning J-M, et al. (2017) Subacute Subclinical Brain Infarctions after Transcatheter Aortic Valve Implantation Negatively Impact Cognitive Function in Long-Term Follow-Up. PLoS ONE 12(1): e0168852. doi:10.1371/journal.pone.0168852

The publisher apologizes for these errors.
