# Supplementary material for: Subacute Subclinical Brain Infarctions after Transcatheter Aortic Valve Implantation Negatively Impact Cognitive Function in Long-Term Follow-Up
Source: PLoS One. 2017 Jan 5;12(1):e0168852. doi: 10.1371/journal.pone.0168852 (PMC5215955; doi:10.1371/journal.pone.0168852)
Supplement: S1 Methods — Transfemoral aortic valve implantation Cranial MRI and imaging analysis. (DOCX) [file pone.0168852.s001.docx]

**Supplementary Methods**

All patients scheduled for TAVI between October 2009 and December 2010 were screened for inclusion. Patients were recruited consecutively. However, only 28 of 97 patients were eligible for repeated MRI protocol, due to death (n=35), pacemaker implantation (n=10), logistical reasons (n=11), patients’ withdrawal due to age, multimorbidity and immobility (n=13). Indication was in concordance with the recent consensus statement. Major study inclusion criteria were: a) severe, symptomatic aortic stenosis with or without regurgitation and high or excessive peri-operative risk, b) echocardiographic aortic valve annulus diameter >20 and <27 mm, and c) diameter of the ascending aorta <45 mm. Exclusion criteria included contraindications to DW-MRI, e.g. permanent pacemaker implantation, claustrophobia, or hemodynamic instability impeding transport to DW-MRI, hypersensitivity or contraindication to post-interventional dual platelet inhibition; sepsis or active endocarditis; bleeding diathesis or coagulopathy; recent cerebrovascular accident; mitral or tricuspid valvular insufficiency (> grade II); left ventricular or atrial thrombus; previous aortic valve replacement; progressive disease with life expectancy <1 year, and inability to give written informed consent. Furthermore, patients with a non-autarkic lifestyle or a psychiatric disorder were excluded. The study protocol was approved by the local institutional review board and followed the Declaration of Helsinki guidelines. Written informed consent was obtained from all patients.

*Transfemoral aortic valve implantation*

Firstly, valvuloplasty of the aortic valve was performed with a 20- to 25-mm balloon catheter under rapid right ventricular pacing. After manual crimping of the balloon-expandable valve (Edwards-Sapien, Edwards Inc., USA) onto a delivery balloon catheter or loading of the self-expandable prosthesis (Third-generation CoreValve® revalving system, Medtronic Inc., USA) into the sheathed delivery system, the prosthesis was advanced into the left ventricle with retrograde passage of the aortic valve. After positioning with the use of fluoroscopic and angiographic guidance, the balloon-expandable stent valve was deployed by balloon inflation under rapid right ventricular pacing at 160 to 220 bpm. The CoreValve® was deployed stepwise and under guidance by several small-volume angiograms without rapid pacing. Percutaneous closure system (Prostar XL, Abbott Inc.) was routinely used for the closure of the 18 F arterial access site. During TAVI, the patients received weight-adjusted intravenous heparin to achieve an activated clotting time of 300 – 350 s for the duration of the procedure. Additionally, 500 mg of acetylsalicylic acid (ASS) and 300 mg clopidogrel-hydrogensulfate were administered. Dual antiplatelet treatment was continued with 100 mg of ASS and 75 mg of clopidogrel for six months followed by ASS monotherapy.

*Cranial MRI and imaging analysis*

All imaging studies were performed either on a 1.5-T (Philips Intera) or a 3-T (Philips Ingenia) whole body MRI system (Philips Healthcare, Best, The Netherlands). The imaging protocol comprised transversal and coronal DWI, transversal T2-weighted turbo spin echo (Turbo spin echo (TSE); 1.5-T: Repetition time (TR) / Echo time (TE): 4800 / 100 ms; 3-T: Repetition time (TR) / Echo time (TE): 3300 / 80 ms) and Fluid Attenuated Inversion Recovery (FLAIR; 1.5-T: TR/TE 6000/120 ms; 3-T: TR/TE 12000/140 ms) sequences. DWI was performed with a spin-echo echo-planar pulse sequence (1.5-T: TE: 78 ms; TR: 2921 ms; matrix: 128 x 256; section thickness: 5 mm; intersection gap: 1 mm; total acquisition time, 21.4 seconds; 3-T: TE: 47 ms; TR: 3866 ms; matrix: 128 x 256; section thickness: 5 mm; intersection gap: 1 mm; total acquisition time: 46.3 seconds) with diffusion sensitization b-values of 0, 500 and 1000 s/mm^2^. Apparent diffusion coefficient (ADC) maps were calculated for all studies to identify findings with restricted diffusion. All follow-up studies used the same sequence protocol in order to assess the presence or absence of a subsequent infarct at the location of the diffusion abnormality.

Pre-existing brain abnormalities (e.g. microangiopathy, infarctions or atrophy) and the appearance of new hyperintense lesions in DWI at baseline were evaluated. Only DWI alterations consistent with embolic lesions were included in the analysis. In contrast, diffuse alterations in the diffusion-weighted image or patterns of watershed ischemia were excluded. Likewise, post-interventional new lesions were determined on the DWI images with maximum contrast between lesion and normal tissue signal. Total WML volumes and DW-MRI volumes were calculated using a combination of two freely available applications. First, extracerebral structures were extracted using the brain extraction tool (BET) from FSL (1,2). Second, volumetric measurements were determined using the semi-automatic open source application ITK-SNAP (3). Total brain volume was determined by using FAST (FMRIB's Automated Segmentation Tool) from FSL (4).

1. Jenkinson M, Beckmann CF, Behrens TE, Woolrich MW, Smith SM. Fsl. NeuroImage. 2012;62(2):782-90.

2. Woolrich MW, Jbabdi S, Patenaude B, Chappell M, Makni S, Behrens T, et al. Bayesian analysis of neuroimaging data in FSL. NeuroImage. 2009;45(1 Suppl):S173-86.

3. Yushkevich PA, Piven J, Hazlett HC, Smith RG, Ho S, Gee JC, et al. User-guided 3D active contour segmentation of anatomical structures: significantly improved efficiency and reliability. NeuroImage. 2006;31(3):1116-28.

4. Zhang, Y. and Brady, M. and Smith, S. Segmentation of brain MR images through a hidden Markov random field model and the expectation-maximization algorithm. IEEE Trans Med Imag, 20(1):45-57, 2001.
